# Modulating macrophage polarization for the enhancement of fracture healing, a systematic review

**DOI:** 10.1016/j.jot.2022.05.004

**Published:** 2022-08-05

**Authors:** Simon Kwoon-Ho Chow, Carissa Hing-Wai Wong, Can Cui, Michelle Meng-Chen Li, Ronald Man Yeung Wong, Wing-Hoi Cheung

**Affiliations:** Department of Orthopaedics and Traumatology, The Chinese University of Hong Kong, Hong Kong SAR, China

**Keywords:** Osteoporotic fracture, Endochondral ossification, Inflammatory response, macrophage polarization, mechanical stimulation, Pathogen-associated molecular patterns (PAMP)

## Abstract

**Background:**

All fracture repairs start with the innate immune system with the inflammatory response known as the inflammatory stage guided and driven by the secretion of chemokine by the ruptured tissue, followed by the sequential recruitment of neutrophils, monocytes and macrophages. These innate immune cells would infiltrate the fracture site and secrete inflammatory cytokines to stimulate recruitment of more immune cells to arrive at the fracture site coordinating subsequent stages of the repair process. In which, subsidence of pro-inflammatory M1 macrophage and transformation to anti-inflammatory M2 macrophages promotes osteogenesis that marks the start of the anabolic endochondral stage.

**Methods:**

Literature search was performed on Pubmed, Embase, and Web of Science databases (last accessed 15th April 2021) using “macrophage AND fracture”. Review was performed in accordance with the Preferred Reporting Items for Systematic Reviews and Meta-Analyses (PRISMA) guideline.

**Results:**

Eleven pre-clinical animal studies out of 429 articles were included in this systematic review according to our inclusion and exclusion criteria. All of which investigated interventions targeting to modulate the acute inflammatory response and macrophage polarization as evident by various markers in association with fracture healing outcomes.

**Conclusion:**

This systematic review summarizes attempts to modulate the innate immune response with focuses on promoting macrophage polarization from M1 to M2 phenotype targeting the enhancement of fracture injury repair. Methods used to achieve the goal may include applications of damage-associated molecular pattern (DAMP), pathogen-associated molecular pattern (PAMP) or mechanical stimulation that hold high translational potentials for clinical application in the near future.

## 1 Introduction

Fracture healing is an elegantly well-orchestrated bone repair process involving overlapping stages of inflammatory stage, the anabolic or endochondral stage, and finally the coupled catabolic or remodeling stage [1]. All fracture repairs start with the innate immune system with the inflammatory response known as the inflammatory stage [2] guided and driven by the secretion of chemokines by the ruptured tissues, followed by the sequential recruitment of neutrophils, monocytes and macrophages [3]. These innate immune cells would infiltrate the fracture site and secrete inflammatory cytokines to stimulate recruitment of more immune cells to arrive at the fracture site coordinating subsequent stages.

Major cytokines involved in fracture healing include TNF- α (tumour necrosis factor-alpha), IL-1 (interleukin-1), IL-6 and IL-10. Each would take unique surge patterns at the very early inflammatory stage and the early callus remodelling stage [4,5]. In animal fracture models, the lack of TNF- α results in the failure of endochondral cartilage resorption [6] and the lack of IL-6 would impair callus maturation and delay healing [7]. Also, enhancing the inflammatory response in fracture healing by the addition of TNF- α led to better mineralization in the fracture callus; this enhancement was abolished by the inhibition of inflammatory response by anti-TNF and various neutrophil recruitment factors [8]. Pro-inflammatory cytokine IL-1β was also well reported to coordinate specific processes at different stages including osteoblasts and mesenchymal stem cells (MSCs) differentiation [9], stimulation of angiogenesis [10], and mediating intramembranous ossification and callus remodelling [4]. After the surge pro-inflammatory cytokines reach peak, the surge of anti-inflammatory cytokine of IL-10 would follow, causing a subsidence of inflammatory phase and the morphological change of the macrophages from pro-inflammatory M1 to M2 [11,12]. Anti-inflammatory M2 macrophages may have direct roles in fracture healing on wound repair and debris scavenging [13]; and subsequently secrete tissue repairing cytokines including IL-10, TGF-beta, BMP-2 and VEGF and MSCs recruitment and differentiation, endochondral ossification, and angiogenesis [14]. Recently, it has been shown by several studies that the deletion of macrophages in mice fracture healing resulted in non-union [12,15,16], thus highlighting the important coordinating role of macrophages in fracture repair. Circulating and resident macrophages are important to fracture healing that are myeloid cells originated from hematopoetic stem cells [17]. Various subtypes of bone residing macrophages or “osteomacs” exist and heavily influenced by the mix of cytokines in the injury site. Although both osteomacs and osteoclasts are both derived my hematopoietic precursors along the myeloid lineage, the theory that osteoclast generation directly descended from osteomacs is yet to be confirmed [18]. Osteomacs are also reported to have direct regulatory roles on bone-forming osteoblast functions [19]. In brieft, due to macrophage plasticity and pleiotropic functions, direct or indirect roles of macrophage in fracture healing could be largely due to the proportions of ‘right” versus ‘wrong’ macrophages [20].

Fracture healing is often compromised, for example, as ageing and sex hormone depletion during the development of osteoporosis both alter systemic inflammatory response [21,22], indicating a difference in the initiating stage of inflammation during osteoporotic fracture healing. Ageing affects the inflammatory response as demonstrated by the distinctive expression patterns of pro-inflammatory markers (TNF- α and IL-6) and anti-inflammatory markers (IL-10) at early and late stages in life [23,24]. At the same time, estrogen has both immunosuppressive and pro-inflammatory effects [25] and the inflammatory response to injury was lowered in ovariectomized animal models [26,27]. TNF- α and IL-6 are both mediated by the levels of estrogen indirectly through Jun or Nuclear factor-kappa-B (NF-κB) pathways, respectively [28]. Therefore, learning to modulate the inflammatory stage by modulating macrophage polarization could be a good therapeutic target for the enhancement of compromised healing of bones. The objective of this systematic review is to gather current knowledge modulating macrophage polarization for improved fracture healing.

## 2 Methods

### 2.1 Search strategy

The literature search was performed on Pubmed, Embase, and Web of Science databases (last access was 15th April 2021) using the search strategy “macrophage AND fracture”. The review was performed in accordance with the Preferred Reporting Items for Systematic Reviews and Meta-Analyses (PRISMA) guideline [29].

### 2.2 Search criteria

Inclusion criteria were: 1) preclinical or clinical studies that investigated the role of macrophage polarisation in bone healing; 2) bone fracture or bone defect animal studies with treatments or interventions; and 3) in vitro studies that addressed macrophage polarisation mechanisms.

Exclusion criteria were: 1) review papers; 2) articles and editorials; 3) conference abstracts; 4) no full-text literature provided; 5) non-English papers; 6) records published >20 years ago (<2001); 7) not fracture-related; 8) not about macrophage polarisation; and 9) medical complications.

### 2.3 Selection of studies

Duplicates were removed before screening of studies took place. Irrelevant papers were first excluded by the screening of title and abstract. Full text articles of remaining records were later retrieved to assess for eligibility based on the inclusion and exclusion criteria. Two reviewers performed the study selection independently, and disagreements were settled by discussion and consensus.

### 2.4 Data extraction

The following information was extracted by reviewers and presented in tables: 1) animal model used; 2) location and type of fracture, and fixation method; 3) radiological and/or histological evidence of fracture healing; 4) evidence of macrophage polarisation; and 5) interventions used in current in vivo and/or in vitro models, and its therapeutic outcomes.

### 2.5 Data analysis

The included studies had high discrepancies in terms of animal species, methodology, key findings and statistical methodology, and hence a meta-analysis was not conducted. Instead, a qualitative review was performed to address findings that explore the relevance between macrophage polarisation and the outcomes of bone fracture healing.

## 3 Results

### 3.1 Results of the search

**Figure 1:**
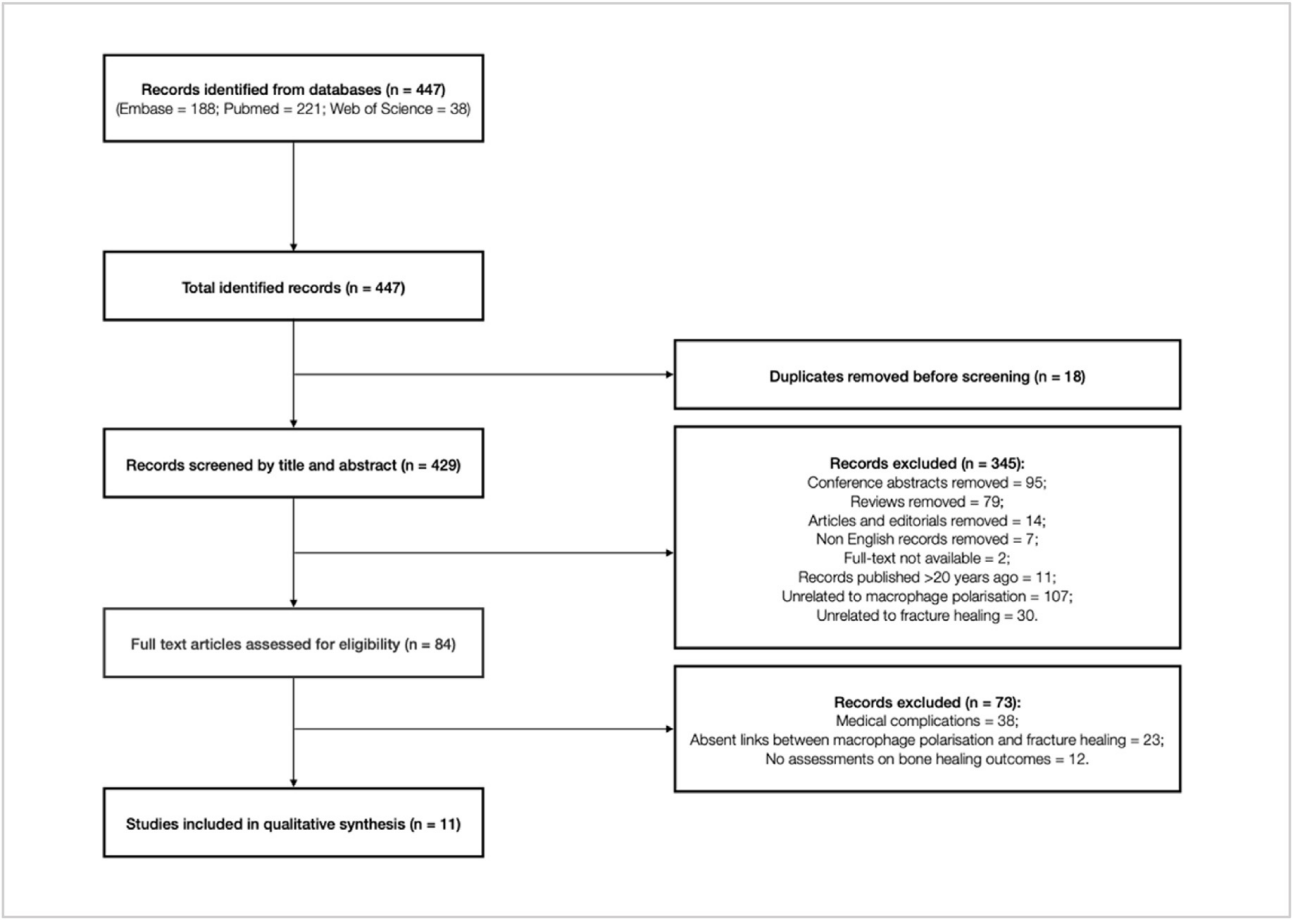
Flowchart showing the process of study selection.

188, 221 and 38 studies were identified from Embase, Pubmed and Web of Science databases respectively, which adds up to a total of 447 records. Duplicated entries were removed (n ​= ​18), leaving 429 studies. We screened the remaining studies by its title and abstract and have further excluded 345 records which have failed to meet the selection criteria. 84 studies were identified for full-text review, and 73 were excluded because of medical complications, absent links between macrophage polarisation and fracture healing, and the lack of bone healing outcomes assessments in the occurrence of macrophage polarisation. Despite attempts to include papers published within the past 20 years, most relevant papers were published within the past 5 years. Our results included a total of 11 manuscripts for analysis [15,16,22,[30], [31], [32], [33], [34], [35], [36], [37]]. The flow diagram shown in Fig. 1 summaries the selection process.

### 3.2 Characteristics of included studies

The selected studies were published from 2016 to 2020. All 11 studies are preclinical studies performed in mice (9 studies) [15,16,22,[30], [31], [32], [33], [34], [35]] and rats (3 studies) [31,36,37] animal models respectively. Study characteristics are summarised in Table 1.

**Table 1:** **Summary of in vivo study characteristics.** ELISA: Enzyme-linked immunosorbent assay; FACS: Fluorescence-activated cell sorting; FTY720: Fingolimod; IL-4/IL-13: The combined treatment of interleukin-4 (IL-4) and IL-13; LMHFV: Low-magnitude high-frequency vibration; Mac1SAP: Mac-1 Sap conjugated antibody; MaR1: Maresin 1; MSR1: Macrophage scavenger receptor 1; PLX3397: Pexidartinib; qPCR: quantitative polymerase chain reaction; 1,25(OH)2D: 1,25-Dihydroxyvitamin D.

First Author, Year	Strain, species, age, gender	Fracture site	Osteotomy type	Fixation	Intervention
Wang, 2016	BALB/c, mice, 10 weeks, M	Tibia	Closed, fracture	Sterile metal pin	FTY720
	Sprague–Dawley, rat, 6–8 weeks, F	Cranial	Open, defect (8 ​mm diameter)	No fixation	
Sandberg, 2017	C57bl6/NRj, mouse, 9 weeks, M	Tibial metaphysis	Open, defect (0.6 ​mm diameter)	0.6 ​mm in diameter custom made titanium screw	Clodronate liposomes
			Open, defect (0.4 ​mm diameter)	No fixation	Clodronate liposomes
Schlundt, 2018	C57BL/6N, mouse, 8 weeks, M	Femoral diaphysis	Closed, fracture	Intramedullary wire	Clodronate ​liposomes
	C57BL/6N, mouse, 12–16 weeks, F	Femoral metaphysis	Open, fracture	Pin (0.45 ​mm diameter), external fixator	IL-4/IL-13
Wasnik, 2018	B6, mouse, 12 weeks, M	Femoral diaphysis	Closed, fracture	30 ​G needle	1,25(OH)2D
Chow, 2019	Sprague–Dawley, rat, 9 months, F	Femoral diaphysis	Closed, fracture	Kirschner wire	LMHFV
Xu, 2019	Sprague–Dawley, rat, N/A, F	Femoral diaphysis	Open, fracture	Kirschner wire	Trehalose
Clark, 2020	C57B6/J, mouse, 3 or 24 months, N/A	Tibia	Closed, fracture	No fixation	PLX3397
Hozain, 2020	Swiss Webster, mouse, 8–10 weeks, F	Femoral diaphysis	Closed, fracture	Intramedullary pins	Mac1SAP toxin
Huang, 2020	C57BL/6J, mouse, 24 months, M&F	Tibia	Open, fracture	Stainless steel pin	MaR1
Zhao, 2020	C57BL/6, mouse, 8 weeks, M	Tibia	Open, defect (0.8 ​mm diameter)	No fixation	Macrophage GIT1 depletion
Zhao, 2020	C57BL/6, mouse, N/A, N/A	Tibia	Open, defect (0.8 ​mm diameter)	No fixation	Macrophage MSR1 knockout

#### 3.2.1 Location, type of fracture and fixation methodology

Of all 11 included studies, 14 treatment models were used to investigate the relationships between fracture healing and macrophage polarisation. 6 of the selected studies adopted a closed fracture model [15,16,22,30,31,37], 3 adopted an open fracture model [16,34,36], and 4 adopted a bone defect model [[31], [32], [33],^35^]. The fracture site location varied among studies, for which 6 studies performed fractures at the tibial shaft [22,[31], [32], [33], [34], [35]], 5 ​at the femoral shaft [15,16,30,36,37], and 1 ​at the cranium [31]. 7 studies used an intramedullary device [15,16,30,31,34,36,37], whereas 2 studies used plate and screws [16,35] for fracture fixation. 5 studies showed no attempts to perform bone fixation at the site of bone injury [22,[31], [32], [33],^35^]. Further details could be found in Table 1.

### 3.3 Macrophage cell markers

Macrophage polarises in response to signals received from the local environment, and differentiates into M1 (inflammatory) and M2 (anti-inflammatory) subtypes to perform different but complementary roles that facilitate bone fracture healing. The selected studies reportedly used cell surface markers, F4/80+ [15,30,32,33,35], F4/80+/CD11b+ [15,30], and CD68+ [16,31,35,37] to separate macrophages from other cell populations. M1 and M2 expressions were identified via additional cell surface markers and measured cytokine expressions, quantified by various approaches and procedures. Inducible nitric oxide synthase (iNOS) was most commonly used by the included studies, and methods such as fluorescence-activated cell sorting (FACS) [30,32,33], quantitative polymerase chain reaction (qPCR) [30,33], and immunostaining of histological sections [15,30,[32], [33], [34],^37^] were employed to measure the expression of this M1 functional marker. Other M1 cell markers, CD80+ [16] and F4/80+/MHCII+/CD86+/CDllb+ [15], were also explored in other studies, alongside M1-associated inflammatory cytokines such as IL-1β, TNF-α, IL-6, IL-1α, oncostatin M (OSM), and IL-12 [30,33,36]. Arginase 1 (Arg-1) and CD206 were the 2 most commonly used M2 functional markers amongst the included studies, detected and measured by FACS [30,33], qPCR [30,33,36], and immunostaining of histological sections [15,16,30,[32], [33], [34],^37^]. Other M2 markers, CD163 [16,33] and IL-10 [36], were also used by studies to quantify M2 macrophage expression. Table 2 summarizes the list of M1 and M2 macrophage markers employed by respective studies.

**Table 2:** Summary of macrophage markers used by the 11 included studies. Arg-1: Arginase 1; FTY720: Fingolimod; IL-1α: Interleukin 1 alpha; IL-1β: Interleukin 1 beta; IL-4/IL-13: The combined treatment of interleukin-4 (IL-4) and IL-13; IL-6: Interleukin 6; IL-10: Interleukin 10; IL-12: Interleukin 12; iNOS: Inducible nitric oxide synthase; LMHFV: Low-magnitude high-frequency vibration; Mac1SAP: Mac-1 Sap conjugated antibody; MaR1: Maresin 1; MHCII: Major histocompatibility complex class II; MSR1: Macrophage scavenger receptor 1; OSM: Oncostatin M; PLX3397: Pexidartinib; TNF-α: Tumour necrosis factor α; 1,25(OH)2D: 1,25-Dihydroxyvitamin D.

First Author, Year	Intervention	Macrophage markers	M1 macrophage markers (or M1 phenotype markers)	M2 macrophage markers (or M2 phenotype markers)
Wang, 2016	FTY720	CD68	N/A	N/A
Sandberg, 2017	Clodronate liposomes	F4/80+, CD68	N/A	N/A
Schlundt, 2018	IL-4/IL-13	CD68	CD80	CD206, CD163
Wasnik, 2018	1,25(OH)2D	F4/80+/CD11b+	IL-1α, IL-1β, OSM, TNF-α, IL-6, IL-12, iNOS	Arg-1
Chow, 2019	LMHFV	CD68	iNOS	CD206
Xu, 2019	Trehalose	N/A	TNF-α, IL-1β, IL-6	Arg-1, IL-10
Clark, 2020	PLX3397	N/A	N/A	N/A
Hozain, 2020	Mac1SAP toxin	F4/80+/CD11b+	F4/80+/MHCII+/CD86+/CDllb+, F4/80+/iNOS+	F4/80+/Arg-1+
Huang, 2020	MaR1	N/A	iNOS	Arg-1
Zhao, 2020	Macrophage GIT1 depletion	F4/80	iNOS	CD206
Zhao, 2020	Macrophage MSR1 knockout	F4/80	iNOS, IL1β	CD206, CD163

### 3.4 Evaluation of employed treatments

Bone fracture healing is a highly complex process, consisting of both metabolic and biological processes. Different treatments were employed by studies to modulate macrophage polarization at various timepoints and correlated with respective bone healing outcomes (details summarized in Tables 3 and 4).

#### 3.4.1 The initial inflammatory stage

Macrophages, alongside other immune factors, are rapidly recruited during the early phase of the initial inflammatory stage and are known for their regulatory role in subsequent bone healing stages. Sandberg et al. treated mice with clodronate liposomes, and reported macrophage depletion 3 days prior to bone defect surgery led to significantly suppressed expression of F4/80 and CD68 pan macrophage markers at day 1 post-injury [35]. The aforementioned changes of macrophage expression were not seen in those treated 2 days prior to surgery at day 3 post-injury.

2 studies evaluated adverse treatments of bone healing, both of which detected a suppression in M1 population during the early pro-inflammatory phase [15,30]. At day 1 post-fracture, Wasnik et al. reported a markedly suppressed M1 and M1-dominated response in mice that have started daily 1,25(OH)2D treatments from the day of fracture induction [30]. This early 1,25(OH)2D treatment also suppressed the expression of osteoblast differentiation associated genes (i.e. runx2, osterix and osteocalcin) and mesenchymal stem cell (MSC) marker genes (i.e. CD90, CD105, and CD73), which suggests that treatment does reduce mice bone healing capabilities. Hozain et al. reported that the administration of Mac-1 Sap conjugated antibody (Mac1SAP) toxin 1 day prior to fracture induction significantly suppressed bone marrow macrophage expression at day 2 post-fracture [15]. This results in a decrease in M1 (F4/80+/MHCII+/CD86+/CDllb+) population, though not statistically significant.

Approaching late inflammatory phase, Schlundt et al. show findings of healthy mice macrophage phenotype polarizing from predominantly M1 to M2 at day 3 post-fracture as M2 macrophages gradually invade the osteotomy gap from surrounding tissues [16]. Wasnik et al. reported a continually suppressed M1-dominated response in mice under early 1,25(OH)2D treatments shown by the reduced IL-1α and IL-1β mRNA expression levels at day 3 post-fracture [30]. Both in vivo and in vitro models also revealed that treatment suppressed the expression of M1 marker iNOS at day 4 post-fracture. Expression of osteoblast differentiation associated genes and MSC marker genes remained suppressed at day 4 post-fracture in mice under early 1,25(OH)2D treatment.

This initial inflammatory stage gradually subsides reaching day 7 post-fracture, and M2 replaces M1 macrophages in mice [16]. 3 studies reported positive bone healing outcomes and M1 suppression at day 7 post-fracture. As reported by Huang et al. M2 anti-inflammatory macrophage population size remained unchanged in aged mice that received MaR1 treatment 3 days post-fracture [34]. The treatment did suppress M1 pro-inflammatory macrophage population size however, suggesting MaR1 treatment is capable of skewing macrophage subtype expressions. Wang et al. reported an accelerated formation and resolution of fracture callus at day 7 post-fracture in Fingolimod (FTY720) treated mice [31], whereas Clark et al. reported significantly enhanced bone formation at day 10 post-fracture in aged mice that received daily Pexidartinib (PLX3397) treatment from 1 day prior to fracture induction [22].

5 studies reported M1 promotion and adverse bone healing outcomes at day 7 post-fracture via interventions of drugs [15,30,35] and knockout treatments [32,33]. Zhao et al. reported an upregulation of M1 marker iNOS and an unchanged expression of M2 marker CD206 at day 7 post-injury in macrophage GIT1 depleted mice via immunofluorescence staining of histological sections [32]. In a separate knockout study, the group identified an enhanced expression of M1-like biomarkers iNOS and IL-1β and suppressed expression of M2 biomarkers CD206 and CD163 in macrophage scavenger receptor 1 (MSR1) knockout mice [33]. They also measured an increase in trabecular spacing (Tb.Sp), formation of less mineralized tissue, and a decrease in bone volume fraction (BV/TV) and trabecular number (Tb.N) in both macrophage GIT1 depleted mice and MSR1 knockout mice, showing that the removal of GIT1 and MSR1 promotes M1 polarization and worsens bone healing outcomes. Wasnik et al. noticed the gradual subsidence of M1 suppression reaching day 7 post-fracture measured in early 1,25(OH)2D treated-mice, though it was also reported that OSM and TNF-α mRNA expressions remained significantly suppressed [30].

Treatment promoted M2 polarization, which was revealed by the augmented expression of M2 marker Arg-1 in vitro and in vivo at day 7 post-fracture. This M2 promotion at day 7 post-fracture is favorable, but not enough to rescue the adverse effects brought by the suppressed M1 response during early inflammatory stage, resulting in poor bone healing outcomes. Hozain et al. reported reduced cartilage formation in Mac1SAP treated mice at day 7 post-fracture via safranin-o staining, though not statistically significant [15]. The depletion of macrophages in mice via clodronate liposome treatments prior to bone injury significantly worsened bone healing outcomes by day 7 post-fracture. Sandberg et al. reported lower BV/TV, bone mineral density (BMD) and maximum pull-out force of screws in mice that received clodronate liposome injections at 1 and 4 days prior to bone defect surgery, in comparison to those treated at day 1 and 3 post-injury [35]. μCT measurements also revealed that mineralized tissues were mainly absent at day 7 post-surgery in mice treated at 1 and 4 days prior to the bone defect surgery, whereas canals were seen to be mostly filled in those treated at day 1 and 3 post-injury. In summary, higher amounts of pro-inflammatory M1 macrophage infiltration detected at the fracture site during the early inflammatory stage (within 3 days) and polarization to anti-inflammatory M2 during the late inflammatory stage (around 7 days) was shown to have a positive effect to the osteoblast differentiation process leading to enhanced capacity of bone regeneration.

#### 3.4.2 The endochondral stage

Reaching the endochondral stage, soft callus and fibrocartilaginous network starts to form as chondrogenesis begins. This cartilaginous callus is later reabsorbed via endochondral ossification at late endochondral phase to form hardened calcified callus in healthy mice. 3 studies reported positive bone healing outcomes during the endochondral stage at day 14 post-fracture. Wang et al. reported that the administration of high doses of FTY720 via ECM gel led to a slight increase in bone volume (BV) at the fracture site compared to mice administered lower doses at day 14 post-fracture [31]. An accelerated formation and enhanced resolution of fracture callus were also observed at day 14 post-fracture in FTY720 treated mice, indicative of enhanced bone healing. The administration of FTY720 via coated graft at the rat cranial defect site led to significant increase in graft vascular density at day 14 post-fracture, showing FTY720 treatment can be effectively delivered via various routes of administration. Huang et al. reported an increased BV and decreased cartilage deposition in calluses of mice administered MaR1 treatment 3 days post-fracture, which again, indicative of enhanced bone healing [34]. Chow et al. observed larger callus volume (CV) at day 14 post-fracture in OVX rats under low-magnitude high-frequency vibration (LMHFV) treatment [37]. It was also reported that fibrin deposition at the fracture site was significantly reduced at day 14 post-fracture, showing enhanced fibrin clearance rate at early stages of fracture healing under LMHFV treatment. Lateral radiographies revealed that callus formation and callus gap bridging capacities were significantly enhanced, and the augmented expression of M2 marker CD206 at day 7 post-fracture has now became weaker at day 14 post-fracture. The combination treatment of non-steroid anti-inflammatory drugs (NSAID) and LMHFV (NSAID/LMHFV) cancels out LMHFV treatment effects, resulting in smaller CV, suppressed callus formation, reduced callus gap bridging capacity, and promoted BV/TV due to suppressed tissue volume (TV) at day 14 post-fracture.

4 studies, including 3 drug treatments [15,16,30] and 1 knockout treatment [33], reported adverse bone healing outcomes at day 14 post-fracture. Wasnik et al. reported a reduction in callus size under early 1,25(OH)2D treatment [30]. Hozain et al. reported larger callus formation, and decreased BV, trabecular volume (Tb.V), trabecular thickness (Tb.Th), and cortical area under Mac1SAP toxin treatment [15]. Zhao et al. reported an increased Th.Sp, reduced BV/TV, and suppressed Tb.N in MSR1 knockout mice, indicating adverse healing outcomes at day 14 post-fracture [33]. The continual depletion of macrophage via clodronate liposome treatment led to significantly suppressed CV and elevated distribution of bone volume within the callus (BV/CV) at day 14 post-fracture as reported by Schlundt et al. [16]. Compact woven bone is observed especially in the peripheral callus regions distant to the fracture gap where bone was formed via intramembranous ossification. A significantly reduced total area of intra-osseous spaces and thus a significantly lowered percentage of bone free area was measured in histological sections, with no significant differences seen in vessel formation at day 14 post-fracture. In summary, the amounts of M2 macrophage determined by the preceding acute inflammatory stage was observed to associate with the altered reparative processes including soft callus formation, fibrin clearance, vascularization of the fracture callus during the endochondral ossification phase.

#### 3.4.3 The coupled remodelling stage

The hard callus gets repeatedly remodelled entering the coupled remodelling stage, which can continue for weeks and even months to fully recover. Balanced activity of osteoclasts and osteoblasts ensure well-governed bone resorption and formation processes which is key to regain normal bone structure at the site of injury. It was reported by Schlundt et al. that a macrophage subtype which was neither CD80 (M1) nor CD206 (M2) positive persisted even after 21 days of healing, suggesting possible macrophage involvements at even later stages of healing [16]. The administration of clodronate liposome treatment led to significant suppression in CV, BV, maximum torsional moment and torsional stiffness at day 21 post-fracture, and collagen II and X expressions were still detectable at this remodelling stage. This indicates that mice without abundant macrophage presence results in delayed maturation of soft callus and impaired endochondral bone formation, which highlights the essential role of macrophage in bone fracture healing. The administration of IL-4/IL-13 treatment during fracture induction in mice led to significant enhancements of both CV and BV at day 21 post-fracture. It's ability to promote M2 polarisation in vitro also confirms the positive effects of an early upregulation of anti-inflammatory signalling during the bone forming process. 2 other studies also reported positive bone healing outcomes at day 21 post-fracture. Chow et al. reported significant enhancement in callus formation seen in LMHFV treated OVX rats at day 21 post-fracture, which effects were cancelled out by NSAID/LMHFV treatment in vivo [37]. Daily administration of PLX3397 from 1 day prior to fracture surgery significantly enhanced bone formation of aged mice at day 21 post-fracture as reported by Clark et al. [22], indicative of improved fracture healing outcomes.

Approaching day 28, 2 mice studies reported positive bone healing outcomes via drug administration of different kinds. Wang et al. reported that mice treated with low doses of FTY720 achieved cortical union [31]. An accelerated formation and resolution of fracture callus was seen at day 28 post-fracture in FTY720 treated mice. Huang et al. recorded an increase in structural stiffness and force to fracture in healing tissues of mice administered MaR1 treatment 3 days after fracture at day 28 post-fracture [34]. 2 other mice studies reported adverse bone healing outcomes at day 28 post-fracture. Wasnik et al. reported reduced bone union, cortex remodelling, BV/TV and Tb.Th under early 1,25(OH)2D treatment, with bigger fracture gaps and fewer bones formed at the site of fracture [30]. Schlundt et al. revealed that clodronate liposome suppressed CV and BV, enhanced BV/CV, and delayed bone mineralisation process indicated by prominent presence of cartilage seen at day 28 post-fracture [16]. The significantly reduced maximum torsional moment and torsional stiffness measured at 28 post-fracture can be explained by the unmineralized fracture gaps seen in treated mice, indicative of major delays in the bone healing process. The reportedly enhanced intramembranous bone formation did not lead to successful bridging of fracture gaps, due to the delayed and suppressed endochondral ossification response.

Both rat studies reported positive bone healing outcomes at day 28 post-fracture, both of which showed an improved disease condition under treatment. Xu et al. reported an elimination of cortical gaps and the formation of bridging callus in trehalose-treated sleep deprived (SD) rats [36]. Trehalose reportedly enhanced BMD, BV/TV and Tb.Th values, and significantly suppressed Tb.Sp values of treated SD rats. Intact cortical bone and nearly normal trabecular bone structure were also formed at the callus area at a reduced level of inflammatory cell infiltration, indicating significant recovery of bone healing capacities under trehalose treatment. Treatment also significantly suppressed the elevated expression of M1 markers in lipopolysaccharides (LPS) induced RAW264.7 ​cells in vitro and in SD rat model in vivo at day 28 post-fracture. The expression of M2 markers Arg-1 and IL-10 were also enhanced by trehalose in vitro, confirming the therapeutic capacity of trehalose to promote M2 polarisation. Chow et al. reported better bridging of callus gaps at day 28 post-fracture in OVX rats under LMHFV treatment via μCT measurements [37]. More bridging of callus gaps and enhancements in callus formation capacity were seen at day 28 post-fracture in LMHFV treated OVX rats via lateral radiographies. NSAID/LMHFV treatment cancels out the LMHFV treatment effects seen in OVX rats, resulting in poorer callus gap bridging and suppressed callus formation capacity.

Approaching day 29 and beyond, only rat studies reported findings on modulated bone healing outcomes at this late remodelling stage. Chow et al. reported an enhanced estimated stiffness at day 56 post-fracture in OVX rats under LMHFV treatment [37]. More callus gap bridging was observed at day 56 post-fracture in LMHFV treated OVX rats via lateral radiographies, which effects were cancelled out by NSAID/LMHFV treatment in vivo. Wang et al. reported a significantly higher bone volume upon FTY720 administration via coated grafts at the rat cranial defect site at day 42 post-injury [31]. A significantly enhanced bone density was also measured at day 70 post-injury via μCT. FTY720 treatment led to formation of less extensive vasculature, promotion of robust tissue ingrowth into the graft, enhancement in mature osteoid formation within both the graft region and void region, promotion of dramatic bone progenitors CD29+ cell infiltration into the graft, and modest reductions in accumulated CD68+ macrophages in the graft region at day 84 post-injury. In summary, the sustained presence of anti-inflammatory M2 macrophage is observed to be associated with processes including maturation of the soft callus or woven bone into fully mineralized lamella bone, differentiations of osteoblast and osteoclasts for the continuous remodelling of the fracture callus.

## 4 Discussion

Fracture repair is largely influenced by the early innate immune response, particularly with respect to the role of macrophages play during the process, as demonstrated by these studies. Generally, these studies have demonstrated a suppressed or compromised innate immune response to fracture repair animal model including macrophage depletion, application of clodronate liposome, vitamin D injection, or the application of NSAID would lead to the impairment of bone regeneration. It might be difficult to generalize if a higher or lower inflammatory response is good for bone regeneration, but it is generally accepted that an optimal inflammatory response is required for the smooth transition from one stage to another stage, where other subset of immune cells would play their respective roles to coordinate the reparative events and influence bone formation progenitors [38]. Learning to modulate macrophage polarization or stimulate the innate immune response to achieve better bone healing outcomes are therefore of clinical importance since medical interventions may often interfere with patients’ immune system.

From this systematic review, we can see that macrophages accumulated at the fracture site during the first week of a fracture event (Day 0 to Day 7 in murine models) could be identified by various surface or functional markers such that researchers may be able to identify them and suggest their functional roles with respect to the healing process [38,39]. In relations to the healing of bone, it is generally believed that the polarization of macrophages from the proinflammatory M1 phenotype (CD80+, iNOS+, F4/80+ in most studies) to anti-inflammatory M2 phenotype (CD206+, Arg-1+ in most studies) marks the transition to the anabolic endochondral stage (Week 4–6 in murine models) followed by the invasion of neo-vasculature and osteoblastic cells for bone formation [16]. It is through the polarization of macrophages that creates this microenvironment enabling cytokine cross-talks between anti-inflammatory M2 macrophages and mesenchymal stem cells (MSC) that drives MSC differentiation into the osteogenic lineage [40]. Nevertheless, it has been observed that in most of the studies, focuses were only on the M1 and M2 macrophages without looking into the specific roles of other immune cells present at the fracture site. In fact, a fracture site is invaded quickly with a vast variety of immune cells including neutrophils, T-cells [41], B-cells, killer cells [14], and dendritic cells (DC) [42] expressing different sets of cytokines to shapes the micro-environment driving the healing to progress to the final coupled remodelling stage (beyond week 6 in murine models) when the fractured bone is being remodelled to its normal physiological state. It has been shown that immature dendritic cells could trans-differentiate into osteoclasts under inflammatory conditions so as to mediate the process of bone destruction [43], dendritic cells-derived interferon-λ1 ameliorated inflammatory bone destruction through inhibiting osteoclastogenesis [44]. It's possible that some of the effects may be in fact attributed to activity of dendritic cells. Yet, the precise delineation of macrophage and dendritic cell function in fracture healing processes is still debatable.

Furthermore, subsets of anti-inflammatory M2 macrophages including the M2a, M2b, M2c based on in vitro induction experiences (with various inflammatory cytokines of TNF-α, IFN-γ, LPS, IL-4, IL-13, IL-1b, TGF-b alone or their combinations) [13,45] that are playing even more specific roles in bone or injury repair have not been discussed in these studies. Their presence at various stages of the healing process in relations to the level of each reparative process including mesenchymal stem cell recruitment, soft callus formation, angiogenesis would be the identified knowledge gap we shall pursue further investigation. Therefore, future studies involving more advanced techniques with high throughput including Fluorescence-activated Cell Sorting (FACS), Single-cell sequencing of RNA or DNA would be much advantageous to quantitatively evaluate the composition of cell types and their relative roles during fracture healing.

The present study also summarized the methods by which researchers have been attempting to modulate macrophage polarization for the enhancement of fracture healing. The methods presented in this review mostly comprised of compounds or drugs that are indicated for other diseases or use. These include trehalose, a sugar containing two glucose molecules originated in the nature from bacteria, fungi or insects with immune modulating effects; PTY720 or Fingolimod is an immunomodulating medication indicated for multiple sclerosis; MaR1 or Maresin-1 is an anti-inflammatory compound derived from macrophage for resolving inflammation; PLX-3397 or Pexidartinib is a colony stimulating factor-1 (CSF-1) inhibitor with anti-inflammatory effects; IL-4/IL-13 are cytokines that are known to promote the polarization of M2 macrophages; and finally vibration treatment is a biophysical intervention with reported positive effects to fracture healing. In terms of the effectiveness of the interventions, M1 suppression treatments administered prior to the inflammatory stage lead to worsened bone healing outcomes [15]. An interesting fact worth mentioning is that depleting macrophages during the inflammatory stage (day 1 and 3) do not impact bone healing outcomes, potentially due to the presence of residual M1 macrophages [35]. Studies supported that daily administration of either M1 suppression or macrophage depletion treatments from pre- or mid-inflammatory stage can impair fracture healing [30,37]. Signs of elevated M1 or suppressed M2 detected by the end of inflammatory stage (day 7) often correlates with adverse healing outcomes [32,33], whereas reversed healing outcomes were resulted under M1 suppression or M2 promotion [34,37]. Our observation from these studies is in agreement with previous reports suggesting that persistent polarization of macrophages to an M1 phenotype during the inflammatory and early anabolic phases may be detrimental to fracture healing [13]. In the clinical perspective, these methods are advantageous over more novel compounds as their safety in use in humans have previously been proven for other indications, and that they can be harnessed to act as damage-associated molecular pattern (DAMP) [46] or pathogen-associated molecular pattern (PAMP, e.g. LPS) [47] to stimulate osteogenesis via immune-modulation [48]. Along the same line, other researchers have also demonstrated success in identifying other immune-modulating compounds derived from various sources that would interact with pattern recognizing receptors on immune cells. One example is the use of gamma-radiation inactivated bacteria for controlled stimulation of toll-like receptor-2 (TLR-2, one of the pattern recognizing receptor) for bone regeneration was shown to be successful in vivo [49]. Although there is only one report on modulating macrophage polarization for the enhancement of fracture healing by means of mechanical stimulation, the vibration treatment is advantageous because it is non-pharmaceutical and very safe for use even immediately after a fracture incident [50]. Overall, there is still great potentials for the exploration of compounds exploiting present knowledge in immunology for the regeneration of bone growth in compromised bone healing patient groups.

One limitation of this study is that only limited number of studies employed a “true” fracture animal model and some studies included employed bone defect models instead. As true fracture healing at different sites (diaphyseal or metaphyseal bone [[51], [52], [53]]) would show different degrees of intramembranous and endochondral ossification process and that would somewhat affect macrophage recruitment. Furthermore, fixation methods and mechanical stability of the fracture model also played an important role in the immune response in various animal models but comparisons systematically conducted with respect to fixation methods. Another limitation is the cell surface markers employed by included studies were not completely consistent between studies or not expressed in humans. Although these markers are largely recognized to generally represent M1 and M2 macrophages, more detailed sub-classifications are emerging to describe even more specific roles of each that these fracture-related studies have not investigated. Finally, although the acute inflammatory response is largely conserved between rodents and humans, more human studies are required to understand and explore on therapeutic potentials of applying modulations.

## 5 Conclusions

The initial inflammatory stage of all bone fracture healing is of paramount importance in coordinating all subsequent stages including angiogenesis, callus formation and remodelling. Modulation of the innate immune response can potentially be applied to enhance compromised fracture healing by exploiting nature's built-in DAMP and PAMP mechanisms or the application of mechanical stimulation. The timing of intervention is also worth investigating to maintain a good population of M1 macrophages during the very early inflammatory stage for subsequent polarization to the M2 macrophages during the anabolic endochondral stage to achieve the desired outcome in terms of fracture healing.

## Declaration of competing interest

The authors have no conflict of interest relevant to this article.
