# Supplementary material for: Modulating macrophage polarization for the enhancement of fracture healing, a systematic review
Source: J Orthop Translat. 2022 Aug 5;36:83–90. doi: 10.1016/j.jot.2022.05.004 (PMC9364046; doi:10.1016/j.jot.2022.05.004)
Supplement: Multimedia component 1 [file mmc1.docx]

**Additional Tables (Large format)**

| **First author, year** | **Intervention** | **Animal Model** | **Phases / Timepoints of Healing** | | | | **Description** |
| --- | --- | --- | --- | --- | --- | --- | --- |
|  |  |  | **Early Inflammatory** | **Late Inflammatory** | **Endochondral** | **Remodelling** |  |
| Zhao, 2020 [32] | Macrophage GIT1 depletion | Mouse |  |  |  |  | Offsprings of GIT1fl/fl mice and Lyz2-Cre mice. |
|  |  |  |  |  |  |  | Less mineralised tissue formation, increased Tb.Sp, decreased BV/TV and Tb.N. Reduced amounts of organised bone regeneration and more filling of connective tissues. |
|  |  |  |  |  |  |  | Promotion of M1-like macrophage, with no significant changes in M2-like macrophages. |
| Zhao, 2020 [33] | Macrophage MSR1 Knockout | Mouse |  |  |  |  | MSR1 KO mice (C57BL/6 background) were acquired. |
|  |  |  |  |  |  |  | Reduced BV/TV, Tb. Th and Tb.N, increased Tb.Sp. |
|  |  |  |  |  |  |  | Reduced BV/TV, Tb.Th and Tb.N, increased Tb.Sp. |
|  |  |  |  |  |  |  | Promotion of M1-like biomarkers and suppression of M2-like biomarkers. |
|  |  |  |  |  |  |  | The promotion of M1-like macrophage fraction and suppression of M2-like macrophage is seen from 3 to 7 days post-surgery. |
| Sandberg, 2017 [35] | Clodronate liposomes | Mouse |  |  |  |  | Groups received injections at either day -4, -1, 1, or 3 respectively, and are harvested at day 7. (0.2 ml single injection) |
|  |  |  |  |  |  |  | Worsen biomechanics properties (BV/TV, BMD, maximum pull-out force of screws) in day -1 and - 4 treated groups, and not in day 1 and 3 treated groups. Mineralised tissues seen in day 1 and 3 treated groups but not day -1 and - 4 treated groups. |
|  | Clodronate liposomes | Mouse |  |  |  |  | Those that received injections at days -3 and harvested at day 1 post-fracture. Those that received injections at day -2 and harvested at day 3 post-fracture. |
|  |  |  |  |  |  |  | Lower expression of F4/80 and CD68 in groups harvested at day 1 post-fracture. |
| Schlundt, 2018 [14] | Clodronate liposome | Mouse |  |  |  |  | Treatment led to a consistent 65% macrophage reduction. Clodronate liposome (100 μl at 5 mg/ml) administered every 5 days starting from 2 days prior to surgery. |
|  |  |  |  |  |  |  | A major delay in healing process from day 7 onwards, with prominent presence of cartilage up to day 28. |
|  |  |  |  |  |  |  | Detections of collagens II and X. |
|  |  |  |  |  |  |  | Reduced CV, higher BV/CV, more compact woven bone formation. |
|  |  |  |  |  |  |  | No fracture union, reduced CV and BV, higher BV/CV, detection of collagens II and X, lower maximum torsional moment and torsional stiffness. |
|  |  |  |  |  |  |  | Un-mineralized fracture gaps with no bone union, higher BV/CV, lower maximum torsional moment and torsional stiffness. |
|  |  |  |  | | | | Clodronate liposome depletes macrophage and changes expression of macrophage and ossification related genes that can impact fracture healing. |
| Wasnik, 2018 [30] | 1,25(  OH)2D | Mouse |  |  |  |  | Local subcutaneous injections administered daily. (100 ng/kg/mouse or 1,000 ng/kg/mouse) |
|  |  |  |  |  |  |  | Weekly x rays collectively shows a delay in fracture repair. |
|  |  |  |  |  |  |  | Reduced callus size |
|  |  |  |  |  |  |  | Reduced bone union, cortex remodeling, BV/TV, Tb.Th. |
|  | 1,25(OH)2D | Mouse |  |  |  |  | Local subcutaneous injections administered daily. (100 ng/kg/mouse or 1,000 ng/kg/mouse) |
|  |  |  |  |  |  |  | Suppression of M1 markers: IL-1β, IL-12, IL-1α, TNFα , IL-6 and OSM |
|  |  |  |  |  |  |  | Suppression of M1 markers: IL-1α, IL-1β |
|  |  |  |  |  |  |  | Suppression of M1 markers: iNOS |
|  |  |  |  |  |  |  | Suppression of M1 markers: TNFα and OSM; and Promotion of M2 markers: Arg-1 |
| Hozain, 2020 [15] | Mac1SAP toxin | Mouse |  |  |  |  | Mice receive single intramedullary injection of 2 μg of Mac1SAP toxin at D-1. |
|  |  |  |  |  |  |  | Less cartilage but not significant. |
|  |  |  |  |  |  |  | Larger callus, decrease in BV, Tb.V, Tb.Th, and cortical area. |
|  |  |  |  |  |  |  | Suppressed M1 (F4/80+/MHCII+/CD86+/CDllb+) subtype population but not significant. |
|  |  |  |  |  |  |  | Suppressed M1 subtype population: F4/80+/MHCII+/CD86+/CDllb+, F4/80+/iNOS+; No change in M2 population: F4/80+/Arg-1+. |
| Chow, 2019 [37] | LMHFV/NSAID | OVX rat |  |  |  |  | OVX rats received NSAID (celecoxib at 4 mg/kg) daily oral garage starting from day -2 till endpoint. They also received LMHFV (35 Hz, 0.3 ×g) treatment starting from 2 days post-fracture, daily for 20 min/d and 5 d/week. |
|  |  |  |  |  |  |  | Showed the lowest callus formation capacity across all time-points via weekly x-ray scans. |
|  |  |  |  |  |  |  | Compared to OVX-VT, OVX-VT-NSAID has higher fibrin deposition, lower TV, higher BV/TV and material BMD. |
|  |  |  |  |  |  |  | Compared to OVX-VT, OVX-VT-NSAID has less callus gaps bridging, lower CV and TV, and higher BV/TV. |
|  |  |  |  |  |  |  | Compared to OVX-VT, OVX-VT-NSAID has less callus gap bridging, higher BV/TV. |
|  |  |  |  |  |  |  | Compared to OVX-VT, OVX-VT-NSAID has less callus gaps bridging. |

**Table 3: Summary of adverse treatment healing outcomes.** Blue: Intervention time; Red: Timepoints showing adverse bone healing outcomes; Yellow: Timepoints showing modulated macrophage expression. Arg-1: Arginase 1; BMD: Bone mineral density; BV: Bone volume; BV/CV: distribution of bone volume within the callus; BV/TV: Bone volume fracture; CV: callus volume; IL-1α: Interleukin 1 alpha; IL-1β: Interleukin 1 beta; IL-6: Interleukin 6; IL-12: Interleukin 12; iNOS: Inducible nitric oxide synthase; LMHFV / NSAID: The combined treatment of Low-magnitude high-frequency vibration (LMHFV) treatment and non-steroid anti-inflammatory drugs (NSAID); Mac1SAP: Mac-1 Sap conjugated antibody; MaR1: Maresin 1; MSR1: Macrophage scavenger receptor 1; MHCII: Major histocompatibility complex class II; OSM: Oncostatin M; OVX: Ovariectomy; OVX-VT-NSAID: ovariectomized rats that received LMHFV / NSAID treatment; Tb.N: Trabecular number; Tb.Sp: Trabecular spacing; Tb.Th: Trabecular thickness; Tb.V: Trabecular volume; TNF-α: Tumour necrosis factor α; TV: Tissue volume; 1,25(OH)_2_D: 1,25-Dihydroxyvitamin D.

| **First author, year** | **Intervention** | **Animal Model** | **Phases / Timepoints of Healing** | | | | **Description** |
| --- | --- | --- | --- | --- | --- | --- | --- |
|  |  |  | **Early Inflammatory** | **Late Inflammatory** | **Endochondral** | **Remodelling** |  |
| Xu, 2019 [36] | Trehalose | Sleep deprived rat |  |  |  |  | Rats injected with trehalose intraperitoneally at 1 g/kg/day since sleep deprived procedure began |
|  |  |  |  |  |  |  | No cortical gap, bridging callus formation with intact cortical bone and nearly normal trabecular bone structure formed at fracture site. Higher BMD, BV/TV and Tb.Th, and lower Tb.Sp. Newborn bones seen at the callus. No obvious inflammatory cell infiltration. |
|  |  |  |  |  |  |  | Suppression of M1 markers: serum TNF-α and IL-1β |
| Wang, 2016 [31] | FTY720 | Mouse |  |  |  |  | FTY720 Administered via ECM gel during surgery. |
|  |  |  |  |  |  |  | Accelerated formation and resolution of the fracture callus during the first 3 weeks. |
|  |  |  |  |  |  |  | Both low and high doses of FTY720 show no significant difference but a trend of increasing BV. |
|  |  |  |  |  |  |  | Low doses of FTY720 achieve cortical union. |
|  | FTY720 | Rat |  |  |  |  | FTY720 delivered in coated grafts during surgery. |
|  |  |  |  |  |  |  | Increases vascular density within the graft relative to control. |
|  |  |  |  |  |  |  | Higher BV. |
|  |  |  |  |  |  |  | Higher bone density. |
|  |  |  |  |  |  |  | Less extensive vasculature, robust tissue growth into the graft, increase formation of mature osteoid within the graft region and void region, dramatic increased CD29+ cell infiltration into the graft region, with mild effect on CD90+ cell number. |
|  |  |  |  |  |  |  | Modest reductions in the accumulation of CD68+ macrophages, and a possible skewing towards M2-like phenotypes as proposed by previous studies. |
| Huang, 2020 [34] | Maresin-1 (MaR1) | Aged mouse |  |  |  |  | MaR1 IP injected at 5 ug/kg on day 3 after surgery. Both day 3 and 0 timepoints were performed but day 0 show no significance in all aspects compared to untreated aged mice therefore are not mentioned. |
|  |  |  |  |  |  |  | Reduced cartilage deposition. |
|  |  |  |  |  |  |  | Increased BV. |
|  |  |  |  |  |  |  | Increased the structural stiffness and the force to fracture of the healed tissue. |
|  |  |  |  |  |  |  | Suppression of M1 marker (iNOS) with no change in M2 marker (Arg-1). |
| Clark, 2020 [22] | PLX3397 | Aged mouse |  |  |  |  | Treatment of PLX3397 was administered from day -1 till endpoint. |
|  |  |  |  |  |  |  | Improved fracture healing outcomes: larger fracture callus, increased BV |
|  |  |  |  |  |  |  | Old mice have increased expression of pro‐inflammatory cytokines and markers of M1 macrophages. Callus macrophages of old mice significantly differentially expressed 1,222 genes as compared to young mice, whereas old mice treated with PLX3397 only significantly differentially expressed 64 genes as compared to young macrophages. |
| Schlundt, 2018 [16] | IL-4 / IL-13 | Mouse |  |  |  |  | Scaffold containing 50 ng of IL-4 / IL-13 inserted upon fracture surgery. |
|  |  |  |  |  |  |  | High CV and BV. |
|  |  |  | N/A | | | | Promotion of M2 in vitro: CD68+/CD206+ |
| Chow, 2019 [37] | LMHFV | OVX rat |  |  |  |  | OVX rats received LMHFV (35 Hz, 0.3 ×g) treatment starting from 2 day after fracture creation. The treatment was given daily for 20 min/d and 5 d/week. |
|  |  |  |  |  |  |  | Enhanced callus formation capacity and low fibrin deposition at site. |
|  |  |  |  |  |  |  | Enhanced callus formation capacity and callus bridging, larger CV, low fibrin deposition at site. |
|  |  |  |  |  |  |  | Enhanced callus formation capacity. |
|  |  |  |  |  |  |  | Enhanced callus formation capacity and callus gaps bridging. |
|  |  |  |  |  |  |  | Enhanced callus gaps bridging and estimated stiffness. |
|  |  |  |  |  |  |  | Promotion of pan macrophage and M2 markers: CD68 and CD206. |

**Table 4: Summary of positive treatment healing outcomes.** Blue: Intervention time; Green: Timepoints showing good bone healing outcomes; Yellow: Timepoints showing modulated macrophage expression. Arg-1: Arginase 1; BMD: Bone mineral density; BV: Bone volume; BV/TV: Bone volume fracture; CV: callus volume; ECM: Extracellular matrix; FTY720: Fingolimod; IL-1β: Interleukin 1 beta; IL-4 / IL-13: The combined treatment of interleukin-4 (IL-4) and IL-13; iNOS: Inducible nitric oxide synthase; LMHFV: Low-magnitude high-frequency vibration treatment; MaR1: Maresin 1; PLX3397: Pexidartinib; Tb.Sp: Trabecular spacing; Tb.Th: Trabecular thickness; TNF-α: Tumour necrosis factor α.
